# 2-Amino­pyridinium *trans*-diaqua­bis­(oxalato-κ^2^ *O*,*O*)chromate(III)

**DOI:** 10.1107/S1600536812040950

**Published:** 2012-10-06

**Authors:** Justin Nenwa, Gouet Bebga, Signé Martin, Michel M. Bélombé, Mohammed Mbarki, Boniface P. T. Fokwa

**Affiliations:** a Department of Inorganic Chemistry, University of Yaounde I, POB 812 Yaounde, Cameroon; b Higher Teacher Training College, POB 47, University of Yaounde 1, Cameroon; c Institut für Anorganische Chemie, RWTH Aachen, D-52056 Aachen, Germany

## Abstract

In the title hybrid salt, (C_5_H_7_N_2_)[Cr(H_2_O)_2_(C_2_O_4_)_2_], the Cr^III^ ion is coordinated in a slightly distorted octa­hedral environment by four O atoms from two oxalate ligands in the equatorial plane and by two water O atoms in the axial sites. The 2-amino­pyridinium cation is disordered over two sets of sites in a 0.800 (7):0.200 (7) ratio. In the crystal, N—H⋯O and O—H⋯O hydrogen bonds connect the components into a three-dimensional network. The crystal studied was an inversion twin with components in a ratio 0.75 (2):0.25 (2).

## Related literature

For general background to the coordination chemistry of oxalates, see: Martin *et al.* (2007 ▶). For the structural characterization of organic–inorganic salts containing the [Cr(H_2_O)_2_(C_2_O_4_)_2_]^−^ anion, see: Bélombé *et al.* (2009 ▶); Nenwa *et al.* (2010 ▶); Chérif *et al.* (2011 ▶); Chérif, Abdelhak *et al.* (2012 ▶); Chérif, Zid *et al.* (2012 ▶).
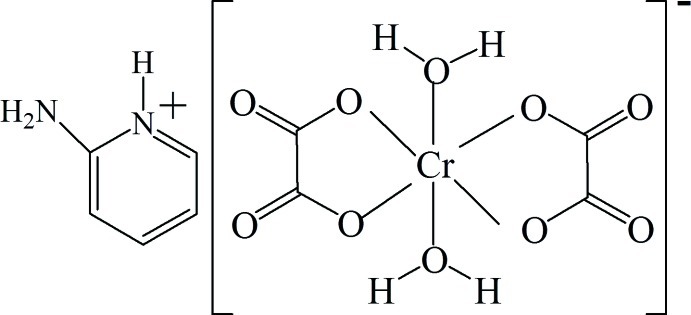


## Experimental

### 

#### Crystal data

(C_5_H_7_N_2_)[Cr(H_2_O)_2_(C_2_O_4_)_2_]
*M* *_r_* = 359.20
Monoclinic, 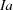

*a* = 6.8627 (14) Å
*b* = 19.434 (4) Å
*c* = 9.854 (2) Å
β = 99.90 (3)°
*V* = 1294.7 (5) Å^3^
*Z* = 4
Mo *K*α radiation
μ = 0.94 mm^−1^
*T* = 100 K
0.23 × 0.15 × 0.10 mm

#### Data collection

Bruker SMART APEX CCD diffractometer
Absorption correction: multi-scan (*SADABS*; Bruker, 2004 ▶) *T*_min_ = 0.811, *T*_max_ = 0.912
9645 measured reflections
3716 independent reflections
3391 reflections with *I* > 2σ(*I*)
*R*_int_ = 0.035

#### Refinement

*R*[*F* ^2^ > 2σ(*F* ^2^)] = 0.039
*wR*(*F* ^2^) = 0.088
*S* = 1.04
3716 reflections
241 parameters
21 restraints
H atoms treated by a mixture of independent and constrained refinement
Δρ_max_ = 0.52 e Å^−3^
Δρ_min_ = −0.30 e Å^−3^
Absolute structure: Flack (1983 ▶), 1793 Friedel pairs
Flack parameter: 0.25 (2)

### 

Data collection: *APEX2* (Bruker, 2004 ▶); cell refinement: *SAINT* (Bruker, 2004 ▶); data reduction: *SAINT*; program(s) used to solve structure: *SHELXS97* (Sheldrick, 2008 ▶); program(s) used to refine structure: *SHELXL97* (Sheldrick, 2008 ▶); molecular graphics: *DIAMOND* (Brandenburg, 2010 ▶); software used to prepare material for publication: *WinGX* Farrugia (1999 ▶).

## Supplementary Material

Crystal structure: contains datablock(s) I, global. DOI: 10.1107/S1600536812040950/lh5534sup1.cif

Structure factors: contains datablock(s) I. DOI: 10.1107/S1600536812040950/lh5534Isup2.hkl

Additional supplementary materials:  crystallographic information; 3D view; checkCIF report

## Figures and Tables

**Table 1:** Hydrogen-bond geometry (Å, °)

*D*—H⋯*A*	*D*—H	H⋯*A*	*D*⋯*A*	*D*—H⋯*A*
O*W*1—H*W*1*A*⋯O23^i^	0.82 (2)	1.86 (2)	2.660 (4)	166 (3)
O*W*1—H*W*1*B*⋯O11^ii^	0.80 (2)	1.92 (2)	2.663 (3)	156 (4)
O*W*2—H*W*2*A*⋯O24^iii^	0.85 (2)	1.78 (2)	2.621 (4)	172 (4)
O*W*2—H*W*2*B*⋯O12^iv^	0.80 (2)	1.95 (2)	2.687 (3)	153 (4)
N1—H1*A*⋯O12^v^	0.88	2.33	3.183 (5)	164
N1—H1*B*⋯O23	0.88	2.38	3.251 (4)	171
N2—H2⋯O13^v^	0.88	2.02	2.865 (3)	159

Symmetry codes: (i) 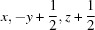; (ii) 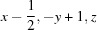; (iii) 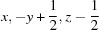; (iv) 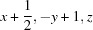; (v) 
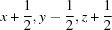
.

**Fig. 1.**
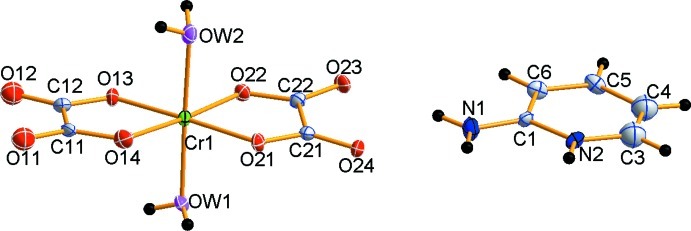
The asymmetric unit of the title compound with displacement ellipsoids drawn drawn at the 50% probability level. The minor component of disorder in the cation is not shown.

**Fig. 2.**
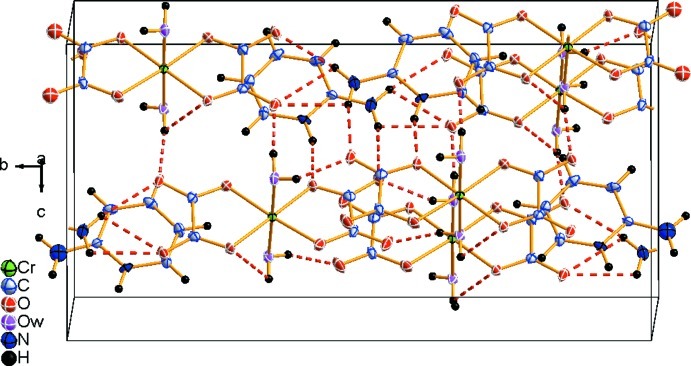
Crystal packing of the title compound, showing the components linked via N—H···O and O—H···O hydrogen bonds (dashed lines) forming a three-dimensional network. The disorder is not shown.

## supplementary crystallographic information

### Comment

The coordination chemistry of oxalates (C_2_O_4_^2-^) continues to receive considerable attention, largely due to the ability of this ion to act as a remarkably flexible ligand system in complexations with a wide range of metal ions (Martin *et al.*, 2007). In the course of recent years, a few organic–inorganic hybrid salts of the form A[Cr(H_2_O)_2_(C_2_O_4_)_2_].xH_2_O (A^+^ = aromatic iminium cation, 0 ≤*x*≤ 1) have been reported (Bélombé *et al.*, 2009; Nenwa *et al.*, 2010; Chérif *et al.*, 2011; Chérif, Abdelhak *et al.*, 2012; Chérif, Zid *et al.*, 2012). These salts form crystal structures for which a set of interesting solid state properties, magnetic interactions, optical and/or optoelectronic effects, or combinations thereof may be expected. With the pyridinium core cation for instance, the resulting salts diversely crystallize in non-centrosymmetric space groups *Fdd2* and *Pna2_1_* (Chérif, Abdelhak *et al.*, 2012, 2011), or in the centrosymmetric space groups *P2_1_/c* (Chérif, Zid *et al.*, 2012) and *C2/c* (Nenwa *et al.*, 2010), depending on the nature of the subtituent and/or the position of substitution. In the present contribution, we report the structure of a homologous salt in the non-centrosymmetric *I2/a* space group.

The asymmetric unit of the title compound is shown in Fig. 1. The main geometrical features of the [C_5_H_7_N_2_]^+^ cation are in agreement with those found in salts with similar cationic entities (Bélombé *et al.*, 2009; Nenwa *et al.*, 2010; Chérif *et al.*, 2011; Chérif, Abdelhak *et al.*, 2012; Chérif, Zid *et al.*, 2012). The Cr^III^ ion adopts a slightly distorted octahedral coordination environment involving four oxalate O atoms (O13, O14, O21, O23) in equatorial sites and two water O atoms (OW1, OW2) in axial sites. The equatorial Cr—O distances are shorter than the axial Cr—O distances. The bond distances in the complex anion (Table 1) are comparable with those reported for the 4-dimethylaminopyridinium compound (Nenwa *et al.*, 2010).

In the crystal structure, intermolecular N—H···O (carbonyl) and O—H···O hydrogen bonds (Table 2, Fig. 2) connect the components into a three-dimensional network.

### Experimental

A mixture of 2-aminopyridine (1 mmol, 100 mg) and oxalic acid (2 mmol, 260 mg) was dissolved in 30 ml of ethanol. An aqueous solution (20 ml) of CrCl_3_.6H_2_O (1 mmol, 266.5 mg) was added in successive small portions and stirred for 4 h continuously. The final blue–violet solution obtained was left at room temperature and crystals suitable for X-ray diffraction were obtained after a few days.

### Refinement

The H atoms were positioned geometrically, with C—H, N—H distances of 0.95 and 0.86 Å respectively, and constrained to ride on their parent atoms, with *U*_iso_(H) = 1.2U_eq_(C,N). The water H atoms were initially located in a difference Fourier map and refined with distance restraints of d(O—H1) = 0.83 (2) with all *U*_iso_(H) values restained to have the same value.

### Crystal data

**Table:**

(C_5_H_7_N_2_)[Cr(H_2_O)_2_(C_2_O_4_)_2_]	*F*(000) = 732
*M**_r_* = 359.20	*D*_x_ = 1.843 Mg m^−^^3^
Monoclinic, *I**a*	Mo *K*α radiation, λ = 0.71073 Å
Hall symbol: I -2ya	Cell parameters from 3716 reflections
*a* = 6.8627 (14) Å	θ = 2.4–30.7°
*b* = 19.434 (4) Å	µ = 0.94 mm^−^^1^
*c* = 9.854 (2) Å	*T* = 100 K
β = 99.90 (3)°	Prism, blue
*V* = 1294.7 (5) Å^3^	0.23 × 0.15 × 0.10 mm
*Z* = 4	

### Data collection

**Table:**

Bruker SMART APEX CCD diffractometer	3716 independent reflections
Radiation source: fine-focus sealed tube	3391 reflections with *I* > 2σ(*I*)
Graphite monochromator	*R*_int_ = 0.035
φ and ω scans	θ_max_ = 30.7°, θ_min_ = 2.4°
Absorption correction: multi-scan (*SADABS*; Bruker, 2004)	*h* = −9→9
*T*_min_ = 0.811, *T*_max_ = 0.912	*k* = −27→26
9645 measured reflections	*l* = −13→13

### Refinement

**Table:**

Refinement on *F*^2^	Secondary atom site location: difference Fourier map
Least-squares matrix: full	Hydrogen site location: inferred from neighbouring sites
*R*[*F*^2^ > 2σ(*F*^2^)] = 0.039	H atoms treated by a mixture of independent and constrained refinement
*wR*(*F*^2^) = 0.088	*w* = 1/[σ^2^(*F*_o_^2^) + (0.0372*P*)^2^ + 2.2822*P*] where *P* = (*F*_o_^2^ + 2*F*_c_^2^)/3
*S* = 1.04	(Δ/σ)_max_ < 0.001
3716 reflections	Δρ_max_ = 0.52 e Å^−^^3^
241 parameters	Δρ_min_ = −0.30 e Å^−^^3^
21 restraints	Absolute structure: Flack (1983), 1793 Friedel pairs
Primary atom site location: structure-invariant direct methods	Flack parameter: 0.25 (2)

### Special details

**Table:**

Experimental. A mixture of 2-aminopyridine (1 mmol, 100 mg) and oxalic acid (2 mmol, 260 mg) was dissolved in 30 ml of ethanol. An aqueous solution (20 ml) of CrCl3.6H2O (1 mmol, 266.5 mg) was added in successive small portions and stirred for 4 h continuously. The final blue-violet solution obtained was left at room temperature and crystals suitable for X-ray diffraction were obtained after a few days.
Geometry. All s.u.'s (except the s.u. in the dihedral angle between two l.s. planes) are estimated using the full covariance matrix. The cell s.u.'s are taken into account individually in the estimation of s.u.'s in distances, angles and torsion angles; correlations between s.u.'s in cell parameters are only used when they are defined by crystal symmetry. An approximate (isotropic) treatment of cell s.u.'s is used for estimating s.u.'s involving l.s. planes.
Refinement. Refinement of *F*^2^ against ALL reflections. The weighted *R*-factor *wR* and goodness of fit *S* are based on *F*^2^, conventional *R*-factors *R* are based on *F*, with *F* set to zero for negative *F*^2^. The threshold expression of *F*^2^ > 2σ(*F*^2^) is used only for calculating *R*-factors(gt) *etc*. and is not relevant to the choice of reflections for refinement. *R*-factors based on *F*^2^ are statistically about twice as large as those based on *F*, and *R*-factors based on ALL data will be even larger.

### Fractional atomic coordinates and isotropic or equivalent isotropic displacement parameters (Å^2^)

**Table:**

	*x*	*y*	*z*	*U*_iso_*/*U*_eq_	Occ. (<1)
Cr1	0.19814 (16)	0.342519 (16)	0.68436 (13)	0.01109 (9)	
C11	0.2455 (5)	0.48129 (16)	0.7396 (4)	0.0177 (7)	
C12	0.1032 (5)	0.47555 (17)	0.6004 (3)	0.0195 (7)	
O11	0.2936 (4)	0.53790 (12)	0.7885 (3)	0.0321 (6)	
O12	0.0359 (4)	0.52679 (12)	0.5348 (3)	0.0282 (6)	
O14	0.3031 (4)	0.42294 (13)	0.7934 (3)	0.0183 (5)	
O13	0.0649 (4)	0.41336 (12)	0.5602 (3)	0.0180 (5)	
C21	0.2715 (5)	0.20807 (17)	0.7621 (3)	0.0135 (6)	
C22	0.1519 (5)	0.20649 (17)	0.6142 (4)	0.0145 (6)	
O22	0.0979 (3)	0.26575 (12)	0.5654 (2)	0.0155 (5)	
O21	0.3152 (3)	0.26885 (12)	0.8085 (2)	0.0134 (5)	
O24	0.3136 (5)	0.15470 (11)	0.8242 (3)	0.0221 (7)	
O23	0.1161 (5)	0.15033 (11)	0.5525 (3)	0.0201 (6)	
OW1	−0.0319 (4)	0.33987 (10)	0.7854 (3)	0.0145 (5)	
OW2	0.4360 (4)	0.34394 (10)	0.5912 (3)	0.0180 (5)	
N1	0.2574 (5)	0.01644 (13)	0.7419 (4)	0.0213 (9)	0.800 (7)
H1A	0.3224	0.0271	0.8241	0.026*	0.800 (7)
H1B	0.2100	0.0492	0.6839	0.026*	0.800 (7)
N2	0.3078 (5)	−0.09776 (18)	0.7994 (3)	0.0141 (8)	0.800 (7)
H2	0.3733	−0.0840	0.8794	0.017*	0.800 (7)
C1	0.2309 (6)	−0.04927 (14)	0.7051 (4)	0.0134 (8)	0.800 (7)
C6	0.1323 (6)	−0.0728 (2)	0.5772 (4)	0.0150 (8)	0.800 (7)
H6	0.0779	−0.0409	0.5080	0.018*	0.800 (7)
C5	0.1146 (6)	−0.1417 (2)	0.5524 (4)	0.0180 (9)	0.800 (7)
H5	0.0494	−0.1574	0.4650	0.022*	0.800 (7)
C4	0.1916 (6)	−0.19022 (15)	0.6546 (4)	0.0161 (9)	0.800 (7)
H4	0.1756	−0.2382	0.6383	0.019*	0.800 (7)
C3	0.2877 (6)	−0.1664 (2)	0.7750 (4)	0.0152 (9)	0.800 (7)
H3	0.3429	−0.1980	0.8447	0.018*	0.800 (7)
N1A	0.161 (2)	0.0179 (4)	0.6157 (15)	0.031 (4)*	0.200 (7)
H1A1	0.2089	0.0479	0.6796	0.037*	0.200 (7)
H1A2	0.0995	0.0323	0.5348	0.037*	0.200 (7)
N2A	0.275 (2)	−0.0690 (6)	0.7682 (10)	0.021 (3)*	0.200 (7)
H2A1	0.3211	−0.0370	0.8285	0.025*	0.200 (7)
C1A	0.180 (2)	−0.0492 (4)	0.6414 (12)	0.011 (3)*	0.200 (7)
C6A	0.101 (2)	−0.1015 (6)	0.5499 (9)	0.007 (3)*	0.200 (7)
H6A	0.0292	−0.0900	0.4617	0.009*	0.200 (7)
C5A	0.129 (3)	−0.1687 (5)	0.5876 (14)	0.024 (5)*	0.200 (7)
H5A	0.0816	−0.2039	0.5234	0.028*	0.200 (7)
C4A	0.227 (2)	−0.1868 (5)	0.7210 (16)	0.020 (4)*	0.200 (7)
H4A	0.2377	−0.2335	0.7498	0.024*	0.200 (7)
C3A	0.303 (2)	−0.1361 (7)	0.8054 (11)	0.018 (4)*	0.200 (7)
H3A	0.3775	−0.1471	0.8930	0.022*	0.200 (7)
HW1A	0.016 (6)	0.3359 (17)	0.867 (2)	0.028*	
HW1B	−0.099 (5)	0.3728 (13)	0.764 (4)	0.028*	
HW2A	0.406 (6)	0.3418 (16)	0.5044 (19)	0.028*	
HW2B	0.485 (5)	0.3815 (12)	0.600 (4)	0.028*	

### Atomic displacement parameters (Å^2^)

**Table:**

	*U*^11^	*U*^22^	*U*^33^	*U*^12^	*U*^13^	*U*^23^
Cr1	0.01435 (16)	0.00763 (14)	0.01064 (15)	0.0002 (2)	0.00028 (11)	0.0000 (2)
C11	0.0265 (17)	0.0098 (12)	0.0212 (14)	−0.0049 (11)	0.0168 (13)	−0.0041 (10)
C12	0.0276 (17)	0.0154 (15)	0.0190 (15)	0.0026 (12)	0.0138 (13)	0.0021 (12)
O11	0.0492 (16)	0.0181 (11)	0.0355 (14)	−0.0159 (10)	0.0257 (12)	−0.0135 (10)
O12	0.0499 (15)	0.0123 (10)	0.0273 (12)	0.0118 (10)	0.0205 (11)	0.0068 (9)
O14	0.0220 (11)	0.0174 (12)	0.0157 (10)	−0.0056 (9)	0.0036 (9)	−0.0046 (9)
O13	0.0271 (13)	0.0100 (11)	0.0181 (11)	0.0048 (8)	0.0071 (10)	0.0029 (8)
C21	0.0142 (13)	0.0143 (15)	0.0134 (13)	0.0026 (11)	0.0067 (10)	0.0014 (11)
C22	0.0155 (14)	0.0128 (14)	0.0167 (14)	−0.0029 (11)	0.0068 (11)	−0.0014 (11)
O22	0.0213 (12)	0.0118 (12)	0.0133 (10)	−0.0012 (8)	0.0029 (9)	−0.0013 (8)
O21	0.0150 (10)	0.0115 (11)	0.0126 (10)	0.0021 (8)	−0.0008 (8)	0.0015 (8)
O24	0.0332 (15)	0.0117 (12)	0.0247 (14)	0.0056 (9)	0.0147 (11)	0.0070 (8)
O23	0.0277 (13)	0.0115 (11)	0.0239 (13)	−0.0059 (9)	0.0121 (10)	−0.0076 (8)
OW1	0.0145 (12)	0.0110 (11)	0.0185 (12)	0.0034 (7)	0.0047 (10)	0.0008 (7)
OW2	0.0208 (14)	0.0133 (12)	0.0198 (12)	−0.0025 (7)	0.0031 (10)	−0.0011 (7)
N1	0.0254 (17)	0.0137 (14)	0.0246 (16)	−0.0009 (11)	0.0033 (13)	−0.0023 (11)
N2	0.0150 (15)	0.0179 (19)	0.0084 (13)	0.0007 (12)	−0.0013 (11)	0.0031 (12)
C1	0.016 (2)	0.0140 (14)	0.011 (2)	0.0005 (12)	0.0032 (15)	0.0028 (12)
C6	0.0124 (17)	0.020 (2)	0.0107 (16)	0.0040 (14)	−0.0026 (13)	−0.0003 (15)
C5	0.0127 (17)	0.024 (2)	0.0158 (18)	0.0029 (16)	−0.0016 (13)	−0.0073 (16)
C4	0.0159 (18)	0.0116 (14)	0.020 (2)	−0.0013 (14)	0.0019 (19)	−0.0036 (12)
C3	0.0151 (18)	0.0116 (17)	0.0193 (19)	−0.0014 (14)	0.0039 (14)	−0.0011 (14)

### Geometric parameters (Å, º)

**Table:**

Cr1—O22	1.949 (2)	N2—C1	1.364 (4)
Cr1—O13	1.960 (2)	N2—H2	0.8800
Cr1—O14	1.962 (2)	C1—C6	1.402 (5)
Cr1—O21	1.963 (2)	C6—C5	1.363 (5)
Cr1—OW2	2.006 (3)	C6—H6	0.9500
Cr1—OW1	2.007 (3)	C5—C4	1.414 (5)
C11—O11	1.223 (4)	C5—H5	0.9500
C11—O14	1.285 (4)	C4—C3	1.337 (5)
C11—C12	1.545 (4)	C4—H4	0.9500
C12—O12	1.233 (4)	C3—H3	0.9500
C12—O13	1.285 (4)	N1A—C1A	1.331 (4)
C21—O24	1.214 (4)	N1A—H1A1	0.8800
C21—O21	1.283 (4)	N1A—H1A2	0.8800
C21—C22	1.545 (3)	N2A—C3A	1.359 (5)
C22—O23	1.253 (4)	N2A—C1A	1.363 (5)
C22—O22	1.278 (4)	N2A—H2A1	0.8800
OW1—HW1A	0.816 (18)	C1A—C6A	1.402 (5)
OW1—HW1B	0.796 (18)	C6A—C5A	1.363 (5)
OW2—HW2A	0.846 (18)	C6A—H6A	0.9500
OW2—HW2B	0.803 (18)	C5A—C4A	1.414 (6)
N1—C1	1.331 (3)	C5A—H5A	0.9500
N1—H1A	0.8800	C4A—C3A	1.337 (6)
N1—H1B	0.8800	C4A—H4A	0.9500
N2—C3	1.358 (4)	C3A—H3A	0.9500
			
O22—Cr1—O13	94.80 (11)	C3—N2—C1	122.9 (3)
O22—Cr1—O14	176.23 (12)	C3—N2—H2	118.6
O13—Cr1—O14	82.55 (9)	C1—N2—H2	118.6
O22—Cr1—O21	83.15 (7)	N1—C1—N2	117.3 (4)
O13—Cr1—O21	176.38 (11)	N1—C1—C6	125.5 (4)
O14—Cr1—O21	99.63 (11)	N2—C1—C6	117.2 (3)
O22—Cr1—OW2	88.05 (9)	C5—C6—C1	119.8 (3)
O13—Cr1—OW2	91.94 (10)	C5—C6—H6	120.1
O14—Cr1—OW2	89.36 (10)	C1—C6—H6	120.1
O21—Cr1—OW2	90.98 (10)	C6—C5—C4	121.1 (3)
O22—Cr1—OW1	93.02 (9)	C6—C5—H5	119.5
O13—Cr1—OW1	90.31 (10)	C4—C5—H5	119.5
O14—Cr1—OW1	89.68 (10)	C3—C4—C5	117.9 (3)
O21—Cr1—OW1	86.82 (9)	C3—C4—H4	121.0
OW2—Cr1—OW1	177.42 (14)	C5—C4—H4	121.0
O11—C11—O14	126.0 (4)	C4—C3—N2	121.1 (3)
O11—C11—C12	120.1 (3)	C4—C3—H3	119.4
O14—C11—C12	113.9 (3)	N2—C3—H3	119.4
O12—C12—O13	124.1 (3)	C1A—N1A—H1A1	120.0
O12—C12—C11	122.0 (3)	C1A—N1A—H1A2	120.0
O13—C12—C11	114.0 (3)	H1A1—N1A—H1A2	120.0
C11—O14—Cr1	114.8 (2)	C3A—N2A—C1A	122.7 (4)
C12—O13—Cr1	114.8 (2)	C3A—N2A—H2A1	118.6
O24—C21—O21	125.9 (3)	C1A—N2A—H2A1	118.6
O24—C21—C22	120.0 (4)	N1A—C1A—N2A	117.8 (5)
O21—C21—C22	114.1 (3)	N1A—C1A—C6A	125.0 (5)
O23—C22—O22	125.6 (4)	N2A—C1A—C6A	117.1 (3)
O23—C22—C21	120.2 (4)	C5A—C6A—C1A	120.0 (4)
O22—C22—C21	114.2 (3)	C5A—C6A—H6A	120.0
C22—O22—Cr1	114.4 (2)	C1A—C6A—H6A	120.0
C21—O21—Cr1	113.8 (2)	C6A—C5A—C4A	120.9 (4)
Cr1—OW1—HW1A	106 (3)	C6A—C5A—H5A	119.6
Cr1—OW1—HW1B	108 (3)	C4A—C5A—H5A	119.6
HW1A—OW1—HW1B	117 (3)	C3A—C4A—C5A	117.8 (4)
Cr1—OW2—HW2A	113 (3)	C3A—C4A—H4A	121.1
Cr1—OW2—HW2B	109 (3)	C5A—C4A—H4A	121.1
HW2A—OW2—HW2B	100 (3)	C4A—C3A—N2A	121.3 (4)
C1—N1—H1A	120.0	C4A—C3A—H3A	119.4
C1—N1—H1B	120.0	N2A—C3A—H3A	119.4
H1A—N1—H1B	120.0		

### Hydrogen-bond geometry (Å, º)

**Table:**

*D*—H···*A*	*D*—H	H···*A*	*D*···*A*	*D*—H···*A*
O*W*1—H*W*1*A*···O23^i^	0.82 (2)	1.86 (2)	2.660 (4)	166 (3)
O*W*1—H*W*1*B*···O11^ii^	0.80 (2)	1.92 (2)	2.663 (3)	156 (4)
O*W*2—H*W*2*A*···O24^iii^	0.85 (2)	1.78 (2)	2.621 (4)	172 (4)
O*W*2—H*W*2*B*···O12^iv^	0.80 (2)	1.95 (2)	2.687 (3)	153 (4)
N1—H1*A*···O12^v^	0.88	2.33	3.183 (5)	164
N1—H1*B*···O23	0.88	2.38	3.251 (4)	171
N2—H2···O13^v^	0.88	2.02	2.865 (3)	159

Symmetry codes: (i) *x*, −*y*+1/2, *z*+1/2; (ii) *x*−1/2, −*y*+1, *z*; (iii) *x*, −*y*+1/2, *z*−1/2; (iv) *x*+1/2, −*y*+1, *z*; (v) *x*+1/2, *y*−1/2, *z*+1/2.
